# Probabilistic Models for Two-Phase Materials

**DOI:** 10.3390/ma18133064

**Published:** 2025-06-27

**Authors:** Mircea Grigoriu

**Affiliations:** School of Civil and Environmental Engineering, Cornell University, Ithaca, NY 14853-3501, USA; mdg12@cornell.edu

**Keywords:** binomial/Poisson points, Gaussian, filtered Poisson fields, level-cut fields, Monte Carlo simulation, two-phase materials, random sets, Voronoi tessellation

## Abstract

Level-cut Gaussian/filtered Poisson, mosaic, and Voronoi tessellation random fields are used to model two-phase random materials. Essential properties of these random fields are reviewed and Monte Carlo algorithms for generating synthetic two-phase materials are presented. Numerical examples are used to illustrate the implementation and features of these models for two-phase materials.

## 1. Introduction

Mechanical properties, geometrical characteristics, and other features of the microstructures of nominally identical material specimens exhibit spatial fluctuations which differ from specimen to specimen. Deterministic models can capture features of single specimens but are incapable of characterizing populations of material specimens. In contrast, probabilistic models can be tuned to match statistics of populations of nominally identical specimens. Their realizations can be viewed as synthetic versions of actual material specimens. For example, the samples of the random field in [1] constitute synthetic concrete aggregates whose statistics are consistent with those obtained from measurements.

Our objective is to discuss probabilistic models for two-phase random materials which are frequently used in applications, rather than present an exhaustive review of these models. The discussion uses results from some of the most cited studies on this topic—the books cited in [2,3,4,5,6] and the papers in [7,8,9,10,11]. Two-phase materials have two components/phases, referred to as matrix and inclusions, e.g., cement paste and aggregates in concrete specimens.

A common feature of the probabilistic models for two-phase random materials considered in this study is that their inclusions have random geometries and locations. However, the way in which these models construct inclusions and two-phase materials differ. For example, the inclusions produced by the level-cut random fields of Section 2 are exceedances of Gaussian and filtered Poisson fields above specified levels. The mosaic fields of Section 3 place random sets (inclusions) at Binomial and Poisson point fields defined on the specimen domain. The Voronoi tessellation fields of Section 4 partition the specimen domain in disjoint subsets which are designated as inclusions with specified probabilities.

The subsequent four sections are dedicated to the three types of probabilistic models for two-phase materials, i.e., level-cut, mosaic, and tessellation random fields. The sections define these models, establish properties of interest in applications, provide Monte Carlo algorithms for generating realizations of these models, i.e., synthetic two-phase material specimens, and present numerical examples illustrating the implementations and the capabilities of these models.

## 2. Level-Cut Random Fields

Consider a real-valued Gaussian/non-Gaussian field {Y(x),x∈D} with continuous realizations defined on a probability space (Ω,F,P), where *D* is a bounded subset of $R^{d}$, d=1,2,3. Let a:D→(0,∞) be a deterministic continuous function. The binary random field

(1) U(x)=1(|Y(x)|>a(x)),x∈D,

referred to as the level-cut field/model, takes the values U(x)=1 if |Y(x)|>a(x) and U(x)=0 otherwise.

It seems that the level-cut model was first proposed in [10] for studying porous media. In our context, the subsets on which *U* is 1 and 0, i.e., the subsets {x∈D:|Y(x)|>a(x)} and {x∈D:|Y(x)|≤a(x)}, define the inclusions and the matrix of random two-phase materials.

The definition in (1) holds for any random field *Y*. The subsequent two subsections construct two-phase material models based on Gaussian and filtered Poisson random fields. A useful discussion on level-cut Gaussian fields can be found in [11,12] (Sect. 8.2). Two-phase materials based on filtered Poisson fields are examined in [8,9].

### 2.1. Gaussian Fields

Suppose that Y(x)=G(x) in (1) is an inhomogeneous Gaussian field with mean, correlation, and covariance functions μ(x)=E[G(x)], r(x,y)=E[G(x)G(y)], and c(x,y)=r(x,y)−μ(x)μ(y), with x,y∈D. The variance function of the field is $\sigma {\left(x\right)}^{2}=c\left(x,x\right)$.

#### 2.1.1. Mean and Correlation Functions of Level-Cut Fields

The mean and the correlation functions of the level-cut field in (1) for the case in which Y(x)=G(x) is a Gaussian field result simply from properties of the Gaussian distribution.

**Property 1.**  *The mean and correlation functions are*

(2) $$E\left[U\left(x\right)\right]=E\left[|G\left(x\right)|>a\left(x\right)\right]=\Phi \left(-\tilde{a}\left(x\right)\right)+\Phi \left(\tilde{b}\left(x\right)\right)$$

*and*

(3) $$\begin{array}{cc} E[U(x)\;U(y)] & =E\left[1\left(|G(x)|>a(x)\right)\;1\left(|G(y)|>a(y)\right)\right] \end{array}$$

(4) $$\begin{array}{cc} \; & \;\;\;\;\;\;\;\;\;\;\;\;\;\;\;\;\;\;\;\;\;=\int_{R^{2}}1(|\mu \left(x\right)+\sigma \left(x\right)\alpha |>a\left(x\right))1(|\mu \left(y\right)+\sigma \left(y\right)\beta |>a\left(y\right))\phi \left(\alpha ,\beta ;\rho \left(x,y\right)\right)\;d\alpha \;d\beta \end{array}$$

*where Φ denotes the distribution of the standard Gaussian variable N(0,1), ϕ(·,·;ρ(x,y)) is the joint density of a standard bivariate Gaussian vector with correlation coefficient ρ(x,y)=c(x,y)/(σ(x)σ(y)), $\tilde{a}\left(x\right)=\left(a\left(x\right)-\mu \left(x\right)\right)/\sigma \left(x\right)$, and $\tilde{b}\left(x\right)=$−(a(x)+μ(x))/σ(x).*

**Proof.**  We have

$$\begin{array}{cc} \left\{|G(x)|>a(x)\right\} & =\{|\mu \left(x\right)+\sigma \left(x\right)\;\tilde{G}\left(x\right)|>a\left(x\right)\}\\ \; & =\left\{\mu \left(x\right)+\sigma \left(x\right)\;\tilde{G}\left(x\right)>a\left(x\right)\right\}\cup \left\{\mu \left(x\right)+\sigma \left(x\right)\;\tilde{G}\left(x\right)\leq -a\left(x\right)\right\}\\ \; & =\left\{\tilde{G}\left(x\right)>\tilde{a}\left(x\right)\right\}\cup \left\{\tilde{G}\left(x\right)<\tilde{b}\left(x\right)\right\} \end{array}$$

with the notation $\tilde{G}\left(x\right)=\left(G\left(x\right)-\mu \left(x\right)\right)/\sigma \left(x\right)$. The expectation of U(x) is

$$E\left[U\left(x\right)\right]=E\left[1\left(|G(x)|>a(x)\right)\right]=P\left(|G(x)|>a(x)\right)=P\left(\left\{\tilde{G}\left(x\right)>\tilde{a}\left(x\right)\right\}\cup \left\{\tilde{G}\left(x\right)<\tilde{b}\left(x\right)\right\}\right),$$

since the random variable $1\left(|G(x)|>a(x)\right)$ is unity with probability $P\left(|G(x)|>a(x)\right)$ and zero otherwise. Its final expression follows since the events $\left\{\tilde{G}\left(x\right)>\tilde{a}\left(x\right)\right\}$ and $\cup \{\tilde{G}\left(x\right)<\tilde{b}\left(x\right)\}$ are disjoint and the equalities $P\left(\tilde{G}\left(x\right)>\tilde{a}\left(x\right)\right)=\Phi \left(-\tilde{a}\left(x\right)\right)$ and $P\left(\tilde{G}\left(x\right)<\tilde{b}\left(x\right)\right)=\Phi \left(\tilde{b}\left(x\right)\right)$.

The correlation function of *U* can be calculated from

(5) $$\begin{array}{cc} E[U(x)U(y)] & =E\left[1\left(|G(x)|>a(x)\right)\;1\left(|G(y)|>a(y)\right)\right] \end{array}$$

(6) $$\begin{array}{cc} \; & \;\;\;\;\;\;\;\;\;\;\;\;\;\;\;\;\;\;\;\;\;\;\;=\int_{R^{2}}1(|\mu \left(x\right)+\sigma \left(x\right)\;\alpha |>a\left(x\right))\;1(|\mu \left(y\right)+\sigma \left(y\right)\;\beta |>a\left(y\right))\;\phi \left(\alpha ,\beta ;\rho \left(x,y\right)\right)\;d\alpha \;d\beta \end{array}$$

by using the fact that $\left(\tilde{G}\left(x\right),\tilde{G}\left(y\right)\right)$ is a standard bivariate Gaussian vector with correlation coefficient ρ(x,y). □

Note that the integral $\int_{D}E\left[U\left(x\right)\right]\;dx$ gives the average volume of the inclusions in *D* so that $\int_{D}E\left[U\left(x\right)\right]\;dx/v\left(D\right)$ is the volume fraction, where v(D) denotes the specimen volume. The subsequent statement considered a special case.

**Property 2.**  *If G is a zero-mean, unit-variance homogeneous Gaussian field and the threshold a(x)=a>0 is space-invariant, the mean and correlation functions of U are*

(7) $$\begin{array}{cc} E[U(x)] & =2\;\Phi (-a)\;\mathrm{and}\; \end{array}$$

(8) $$\begin{array}{cc} E[U(x)U(y)] & =\int_{|\alpha |>a,\;|\beta |>a}\phi \left(\alpha ,\beta ;\rho \left(x,y\right)\right)\;d\alpha \;d\beta . \end{array}$$

**Proof.**  The above formula results directly from Property 1. Note that the expectation coincides with the probability $P\left(|G(x)|>a\right)$ and that the correlation function is equal to the volume of the joint density of (G(x),G(y)) under the integration domain. □

#### 2.1.2. Inclusion Properties

The random number N(D) of inclusions in *D* is equal to that of the local maxima of |G(x)| which exceed a(x), i.e., the number of the maxima of G(x) larger than a(x) and the minima of G(x) smaller than −a(x).

The random field *G* has a local maxima exceeding a(x) at x∈D if (1) the field exceeds the level a(x) at this location, i.e., G(x)>a(x), (2) the components of the gradient $G^{\prime }\left(x\right)$ of G(x) vanish, i.e., $\partial G\left(x\right)/\partial x_{i}=0,i=1,\ldots ,d$, and (3) the matrix $G^{\prime \prime }\left(x\right)$ with entries $\partial^{2}G\left(x\right)/\left(\partial x_{i}\;\partial x_{j}\right)\leq 0,i,j=1,\ldots ,d$, is negative definite [2] (Chap. 6). The characterization of N(D) or even its expectation E[N(D)] poses notable difficulties due to the complex dependence of this random number on the properties of the underlying Gaussian field G(x) and the level function a(x).

Relatively simple formulas are only available for the average of the number $N_{0}\left(D\right)$ of local maxima of zero-mean, unit-variance homogeneous Gaussian fields in *D*. The expected value of these maxima can be calculated from

(9) $$E\left[N_{0}\left(D\right)\right]=v\left(D\right)\int_{a}^{\infty }\int \left|\det(\xi^{\prime \prime })\right|\phi \left(\xi ,0,\xi^{\prime \prime }\right)\;d\xi \;d\xi^{\prime \prime }$$

where v(D) denotes the volume of $D,\left(\xi ,\xi^{\prime },\xi^{\prime \prime }\right)$ denote possible values of the Gaussian vector $\left(G\left(x\right),G^{\prime }\left(x\right),G^{\prime \prime }\left(x\right)\right)$ with joint density ϕ(·,·,·), and the second integral is performed over the subset of $R^{d(d+1)/2}$ in which $\xi^{\prime \prime }$ is negative definite. The calculation of $E[N_{0}\left(D\right)]$ is computationally demanding even in this special case. An analytical expression is available for the average number of local maxima of homogeneous Gaussian fields defined on $R^{2}$ which has almost surely continuous partial derivatives up to the second order [2] (Theorem 6.1.2).

Numerical methods need to be employed to (1) estimate the average number E[N(D)] of local maxima of |G(x)| exceeding the threshold a(x), i.e., the average number of generated inclusions in *D*, and (2) characterize the geometry of the resulting inclusions, which depends in a complex manner on the correlation function of G(x) and the threshold function a(x).

**Property 3.**  *Under the assumptions in Property 2, the volume fraction $V_{I}=\int_{D}U\left(x\right)\;dx/v\left(D\right)$ of the inclusions in D is a random variable with mean and variance*

(10) $$\begin{array}{cc} E[V_{I}] & =2\;\Phi (-a)\;\mathrm{and}\\ \mathrm{Var}[V_{I}] & =\frac{1}{v{\left(D\right)}^{2}}\;\int_{D^{2}}\left[\int_{|\alpha |>a,\;|\beta |>a}\phi \left(\alpha ,\beta ;\rho \left(x,y\right)\right)\;d\alpha \;d\beta \right]\;dx\;dy-4\;\Phi {\left(-a\right)}^{2}. \end{array}$$

**Proof.**  The expectation of the volume $I=\int_{D}U\left(x\right)\;dx$ of inclusions in *D* is

$$E[I]=\int_{D}E\left[U(x)\right]\;dx=\int_{D}P\left(|G(x)|>a\right)=2\Phi \left(-a\right)\;v\left(D\right),$$

where the first equality holds by Fubini’s theorem [13] (Sect. 2.8) and the second equality by using the fact that U(x) is a random variable taking the values 1 and 0 with probabilities $P\left(|G(x)|>a\right)$ and $P\left(|G(x)|<a\right)$. Similar arguments give

$$E\left[I^{2}\right]=\int_{D^{2}}E\left[U(x)\;U(Y)\right]\;dx\;dy=\int_{D^{2}}\left[\int_{|\alpha |>a,\;|\beta |>a}\phi \left(\alpha ,\beta ;\rho \left(x,y\right)\right)\;d\alpha \;d\beta \right]\;dx\;dy.$$

The mean and covariance functions in (10) result from the equalities $E\left[V_{I}\right]=E\left[I\right]/v\left(D\right)$, $E\left[V_{I}^{2}\right]=E\left[I^{2}\right]/v{\left(D\right)}^{2}$ and $\mathrm{Var}\left[V_{I}\right]=E\left[V_{I}^{2}\right]-E{\left[V_{I}\right]}^{2}$. □

Consider the limit cases in which the Gaussian process *G* is perfectly correlated and totally uncorrelated (white noise), i.e., ρ(x,y)=1 and ρ(x,y)=0 for all x,y∈D. If ρ(x,y)=1, x,y∈D, then (10) gives $\mathrm{Var}\left[V_{I}\right]=2\;\Phi \left(-a\right)\;\left(1-2\;\Phi (-a)\right)$ since ϕ(α,β;ρ(x,y))=ϕ(α)δ(α−β). The result also follows from the observation that the realizations of *G* are constant so that $V_{I}$ takes the values 1 and 0 with probabilities 2Φ(−a) and Φ(a)−Φ(−a)=1−2Φ(−a). The first two moments of this random variable are $E\left[V_{I}\right]=E\left[V_{I}^{2}\right]=2\;\Phi \left(-a\right)$ so that $\mathrm{Var}\left[V_{I}\right]=2\;\Phi \left(-a\right)\;\left(1-2\;\Phi (-a)\right)$.

If ρ(x,y)=0, x,y∈D, then ϕ(α,β;ρ(x,y))=ϕ(α)ϕ(β) so that $E\left[V_{I}\right]=2\;\Phi \left(-a\right)$, $E\left[V_{I}^{2}\right]={\left(2\;\Phi (-a)\right)}^{2}$ and $\mathrm{Var}[V_{I}]=0$ so that $V_{I}$ is deterministic with value $V_{i}=E\left[V_{I}\right]$. This limit case is equivalent to that in which the material properties are described by ergodic random fields and the specimen size is infinite. Since $E\left[V_{I}\right]=V_{I}$, $V_{I}$ is referred to as the effective volume fraction.

If ρ(x,y)≠0, the volume fraction is a random variable (non-zero variance) which is referred to as the apparent volume fraction [4] (Sect. 7.2.3). The practical implication is that measurements of the volume fraction differ from specimen to specimen.

#### 2.1.3. Monte Carlo Algorithm

It was noted in Section 2.1.2 that there are no practical analytical methods for characterizing inclusions generated by level-cut Gaussian fields. Numerical methods need to be employed. The following three-step Monte Carlo simulation algorithm can be used to construct and characterize synthetic two-phase material specimens occupying a bounded subset *D* of $R^{d}$.

– *Step 1:* Generate samples of the Gaussian field {G(x),x∈D} by, e.g., one of the following two methods. The first represents {G(x),x∈D} by its values $\left\{G\left(x_{i}\right)\right\}$ of the nodes $\left\{x_{i}\right\}$ of a dense mesh in *D* and generates samples of the resulting Gaussian vector by standard algorithms [13] (Sect. 5.2.1). The second method uses the spectral representation or related methods to generate directly realizations of {G(x),x∈D} [13] (Sect. 5.3.1). A simpler version of this method is used in the subsequent section.
– *Step 2:* Identify the subsets of the realization of {G(x),x∈D} whose absolute values exceed specified levels {a(x),x∈D} and record the numbers, the volume, and other geometrical features of the generated subsets (inclusions).
– *Step 3:* Estimate statistics of interest for the inclusions recorded in the previous step.

The algorithm can provide complete information on the generated synthetic two-phase materials. The required number of samples of {G(x),x∈D} depends on the objective. For example, the estimates of the average number of inclusions require by far fewer Gaussian samples than the estimates of, e.g., the geometry of inclusions.

#### 2.1.4. Two-Phase Synthetic Material Specimens

As noted, the number N(D) and the geometry of the level-cut inclusions depend on the correlation function of G(x) and the level function a(x). The complexity of this dependence prevents the development of quantitative relationships between these functions. Only qualitative relationships can be developed.

For example, consider the special case of a zero-mean, unit-variance homogeneous Gaussian field G(x) and a constant level function a(x)=a. Then, N(D) is likely to be small if *G* is strongly correlated since its realizations would be nearly space-invariant. On the other hand, N(D) is expected to be large if *G* is weakly correlated since its realization would exhibit rapid fluctuations which have numerous local maxima. These qualitative observations are illustrated by the following two examples.

Let $\left\{Y_{i}\right\}$, i=1,2, be independent zero-mean, unit-variance stationary Gaussian processes with correlation functions $\rho_{i}\left(u\right)=E\left[Y_{i}\left(x_{i}\right)\;Y_{i}\left(x_{i}+u\right)\right]=\left(1+\lambda_{i}\left|u\right|\right)\exp\left(-\lambda_{i}\left|u\right|\right)$, $\lambda_{i}>0$, defined on the bounded intervals $\left[0,a_{i}\right],a_{i}>0$. Then, $G\left(x_{1},x_{2}\right)=Y_{1}\left(x_{1}\right)+Y_{2}\left(x_{2}\right)$, $x=\left(x_{1},x_{2}\right)\in D$, is a real-valued Gaussian random field defined on the two-dimensional rectangle $D=\left[0,a_{1}\right]\times \left[0,a_{2}\right]$ with mean $E\left[G\left(x\right)\right]=E\left[Y_{1}\left(x_{1}\right)\right]+E\left[Y_{2}\left(x_{2}\right)\right]=0$, correlation function $E\left[G\left(x\right)G\left(x^{\prime }\right)\right]=E\left[Y_{1}\left(x_{1}\right)Y_{1}\left(x_{1}^{\prime }\right)\right]+E\left[Y_{2}\left(x_{2}\right)Y_{2}\left(x_{2}^{\prime }\right)\right]=\rho_{1}\left(x_{1}-x_{1}^{\prime }\right)+\rho_{2}\left(x_{2}-x_{2}^{\prime }\right)$, and variance $E\left[G{\left(x\right)}^{2}\right]=\rho_{1}\left(0\right)+\rho_{2}\left(0\right)=2$, where $x=(x_{1},x_{2})$ and $x^{\prime }=\left(x_{1}^{\prime },x_{2}^{\prime }\right)$.

We use a smoother version $G_{q}\left(x\right)=Y_{1,q}\left(x_{i}\right)+Y_{2,q}\left(x_{i}\right)$ of G(x) by replacing the random processes $Y_{i}$ with truncated Karhunen–Loève representations $Y_{i,q}\left(x_{i}\right)=\sum_{k=1}^{q}Z_{i,k}\;\varphi_{i,k}\left(x_{i}\right)$, i=1,2, where $\left\{\varphi_{i,k}\right\}$ and $\left\{\lambda_{i,k}\right\}$ denote the eigenfunctions and eigenvalues of the correlation functions of $Y_{i}\left(x_{i}\right)$ in $\left[0,a_{i}\right]$ and $\left\{Z_{i,k}\right\}$ are independent zero-mean Gaussian variables with variances $\left\{\lambda_{i,k}\right\}$. We construct the level-cut random field in (1) for $Y\left(x\right)=G_{q}\left(x\right)$ and a space-invariant threshold a(x)=a.

**Figure 1:**
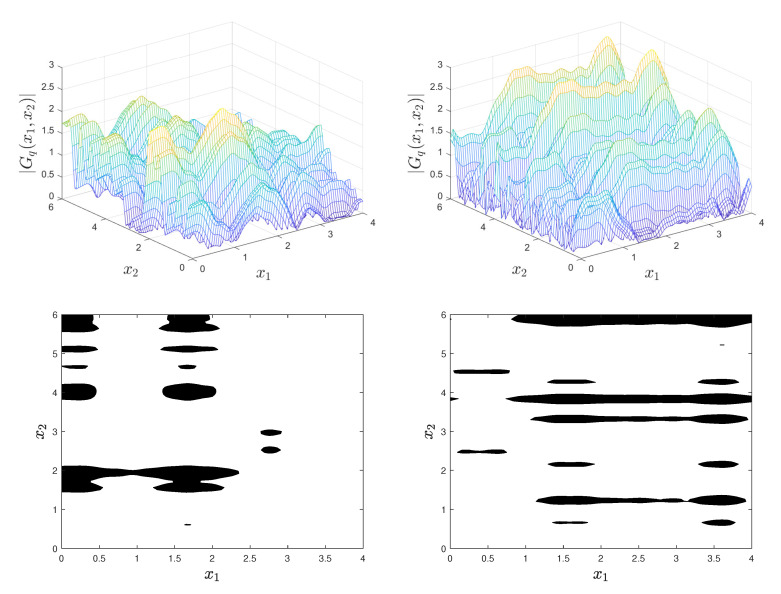
Two sample paths of $\left|G_{q}\right|$ (top left and right panels) and the corresponding inclusions (bottom left and right panels) for $D=\left[0,4\right]\times \left[0,6\right],q=5,\lambda_{1}=2,\lambda_{2}=20$, and a=1.6.

**Figure 2:**
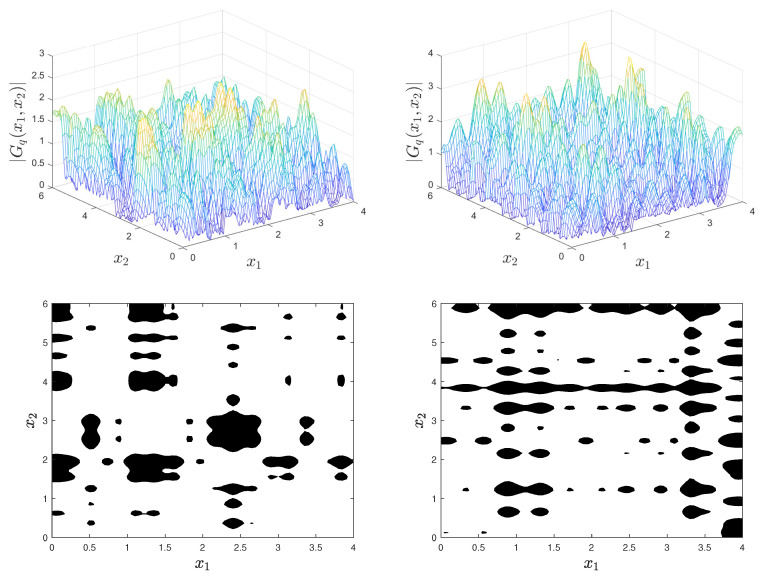
Two sample paths of $\left|G_{q}\right|$ (top left and right panels) and the corresponding inclusions (bottom left and right panels) for $D=\left[0,4\right]\times \left[0,6\right],q=5,\lambda_{1}=10,\lambda_{2}=20$, and a=1.4.

The plots of Figure 1 are for D=[0,4]×[0,6], q=5, $\lambda_{1}=3$, $\lambda_{2}=20$, and a=1.6. The top left and right panels are two independent realizations of $G_{d}$ and the bottom left and right panels are the corresponding realizations of the level-cut random field $1\left(\left|G_{d}\left(x\right)\right|>a\right)$. Similar plots are in Fig. 2 for $D=\left[0,4\right]\times \left[0,6\right],d=5,\lambda_{1}=10,\lambda_{2}=20$, and a=1.4.

The plots in Figure 1 and Figure 2 illustrate qualitatively the dependence of the inclusion shapes and locations on the correlation function of the underlying Gaussian field. For example, the inclusions have small dimensions in the $x_{2}$-direction since the Gaussian fields $G_{q}$ fluctuate rapidly in this direction $\left(\lambda_{2}=20\right)$.

**Figure 3:**
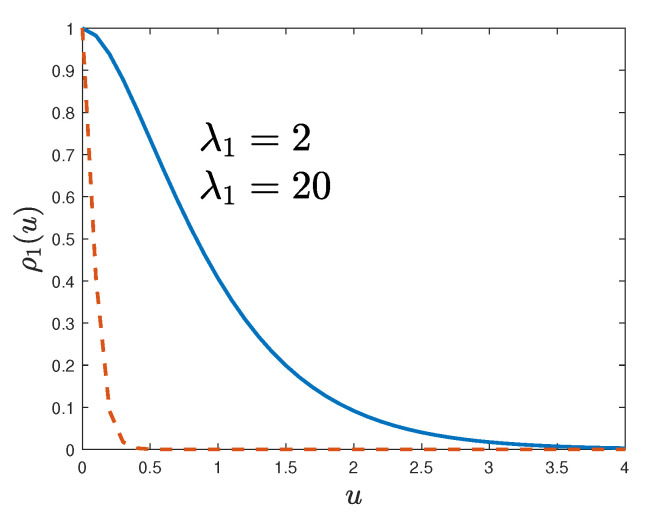
Correlation function $\rho_{1}\left(u\right)$ of $Y_{1,q}$ in the $x_{1}$ direction for $\lambda_{1}=2$ and $\lambda_{1}=10$ (solid and dashed lines).

Figure 3 shows with solid and dashed lines the correlation functions $\rho_{1}\left(u\right)$ of $Y_{1,q}$ for $\lambda_{1}=2$ and $\lambda_{1}=10$. Due to the strong correlation of $Y_{1,q}$ in Figure 1, the Gaussian field $G_{q}$ varies slowly in the $x_{1}$-direction and has few local maxima. This results in elongated inclusions along this coordinate. In contrast, the realizations of $G_{q}$ in Figure 2 vary rapidly in the $x_{1}$-direction due to the weak correlation in this direction and exhibit numerous local maxima and small inclusions.

### 2.2. Filtered Poison Fields

Suppose that Y(x) in (1) is the filtered Poisson field

(11) $$Y\left(x\right)=\sum_{k=1}^{N\left(D^{\prime }\right)}Y_{k}\;h\left(R_{k}\left(x-\Gamma_{k}\right)\right),\;x\in D,$$

where *N* is an inhomogeneous Poisson field with intensity λ(x), $x\in D^{\prime }$, $D^{\prime }⊃D$ is a bounded subset of $R^{d}$, $\left\{\Gamma_{k}\right\}$ denotes Poisson points in $D^{\prime }$, $\left\{Y_{k}\right\}$ denotes independent identically distributed (iid) non-negative random variables, $\left\{R_{k}\right\}$ gives iid (d,d)-rotation matrices, and $h:R^{d}\to \left[0,\infty \right)$ is a deterministic kernel taking its largest value at $x=\Gamma_{k}$ and decreasing with the distance between *x* and $\Gamma_{k}$, e.g., $h\left(\xi \right)=\exp\left(-\sum_{i=1}^{d}{\left(\xi_{i}/\alpha_{i}\right)}^{2}\right)$, where $\alpha_{i}>0$ and $\xi =\left(\xi_{1},\ldots ,\xi_{d}\right)\in R^{d}$. The random elements $\left\{\Gamma_{k}\right\},\left\{Y_{k}\right\}$ and $\left\{R_{k}\right\}$ are mutually independent.

The realizations of *Y* are sums of kernels $\left\{Y_{k}\;h\left(R_{k}\left(x-\Gamma_{k}\right)\right)\right\}$ centered on the Poisson points $\left\{\Gamma_{k}\right\}$ with random orientations defined by the matrices $\left\{R_{k}\right\}$ and heights given by the random variables $\left\{Y_{k}\right\}$. The definition in (11) uses Poisson points in $D^{\prime }⊃D$ since kernels with centers outside *D* can contribute to the values of *Y* in *D*.

The subsequent subsections give properties of the level-cut field in (1) corresponding to *Y* in (11), i.e., the binary random field U(x)=1(|Y(x)|>a(x))=1(Y(x)>a(x)). The latter definition of *U* holds since the realizations of *Y* are non-negative by construction.

#### 2.2.1. Mean and Correlation Functions of Filtered Poisson Fields

Let *N* denote an inhomogeneous Poisson field with intensity λ(x), $x\in R^{d}$. The probability that there are *n* Poisson points in $D^{\prime }$ has the expression

(12) $$P\left(N\left(D^{\prime }\right)=n\right)=\frac{{\left(\int_{D^{\prime }}\lambda \left(x\right)\;dx\right)}^{n}}{n!}\exp\left(-\int_{D^{\prime }}\lambda \left(x\right)\;dx\right),\;n=0,1,\ldots ,$$

since $N\left(D^{\prime }\right)$ is a Poisson random variable with intensity $\int_{D^{\prime }}\lambda \left(x\right)dx$ [14]. There is no Poisson point in $D^{\prime }$ with probability $P\left(N(D^{\prime })=0\right)=\exp\left(-\int_{D^{\prime }}\lambda \left(x\right)\;dx\right)$. The average number of Poisson points in $D^{\prime }$ is given by the integral $E\left[N(D^{\prime })\right]=\int_{D^{\prime }}\lambda \left(x\right)dx$.

If the Poisson field *N* is homogeneous, then λ(x)=λ is constant so that the average number of Poisson points in $D^{\prime }$ is $\lambda \;v(D^{\prime })$, where $v(D^{\prime })$ denotes the volume of $D^{\prime }$. There are on average λ Poisson points per unit volume.

**Property 4.**  *The random vector $\Gamma =\left(\Gamma_{1},\ldots ,\Gamma_{N\left(D^{\prime }\right)}\right)$ conditional on $\left(N\left(D^{\prime }\right)=n\right)$ has joint density*

(13) $$f_{\Gamma |(N\left(D^{\prime })=n)\right)}\left(\xi_{1},\ldots ,\xi_{n}\right)=\frac{\prod_{i=1}^{n}\lambda \left(\xi_{i}\right)}{{\left(\int_{D^{\prime }}\lambda \left(x\right)\;dx\right)}^{n}}.$$

*If N is a homogeneous Poisson field with intensity λ(x)=λ, then $f_{\Gamma |N\left(D^{\prime }\right)}\left(\xi_{1},\ldots ,\xi_{n}\right)=$$\lambda^{n}/{\left(\lambda \;v(D^{\prime })\right)}^{n}=1/v{\left(D^{\prime }\right)}^{n}$.*

**Proof.**  Let $\left\{B_{k}\right\}$ be *n* disjoint bounded subsets of $D^{\prime }$ and set $D^{*}=D^{\prime }\setminus \cup_{k=1}^{n}B_{k}$, e.g., $\left\{B_{k}\right\}$ and $D^{*}$ can be viewed as inclusions and the matrix in $D^{\prime }$. The probability that the numbers $\left\{N\left(B_{k}\right)\right\}$ and $N\left(D^{*}\right)$ of Poisson points in $\left\{B_{k}\right\}$ and $D^{*}$ are $\left\{n_{k}\right\}$ and *m* has the form

$$\begin{array}{cc} \; & P\left(\cap_{k=1}^{n}\left\{N\left(B_{k}\right)=n_{k}\right\}\cap \left\{N\left(D^{*}\right)=m\right\}\right)\\ \; & =\prod_{k=1}^{n}\frac{{\left(\int_{B_{k}}\lambda \left(x\right)\;dx\right)}^{n_{k}}}{n_{k}!}\;\exp\left(-\int_{B_{k}}\lambda \left(x\right)\;dx\right)\frac{{\left(\int_{D^{*}}\lambda \left(x\right)\;dx\right)}^{m}}{m!}\;\exp\left(-\int_{D^{*}}\lambda \left(x\right)\;dx\right)\\ \; & =\exp\left(-\int_{D^{\prime }}\lambda \left(x\right)\;dx\right)\;\frac{{\left(\int_{D^{*}}\lambda \left(x\right)\;dx\right)}^{m}}{m!}\;\prod_{k=1}^{n}\frac{{\left(\int_{B_{k}}\lambda \left(x\right),dx\right)}^{n_{k}}}{n_{k}!} \end{array}$$

by using (13) and the independence of the Poisson random variables $\left\{N(B_{k})\right\}$ and $N(D^{*})$. Then, the probability of having one Poisson point in each $\left\{B_{k}\right\}$ and none in $D^{*}$ is

$$P\left(\cap_{k=1}^{n}\left\{N\left(B_{k}\right)=1\right\}\cap \left\{N\left(D^{*}\right)=0\right\}\right)=\exp\left(-\int_{D^{\prime }}\lambda \left(x\right)\;dx\right)\prod_{k=1}^{n}\int_{B_{k}}\lambda \left(x\right)\;dx$$

so that the probability of $\cap_{k=1}^{n}\left\{N\left(B_{k}\right)=1\right\}\cap \left\{N\left(D^{*}\right)=0\right\}$ conditional on $\left(N(D^{\prime })=n\right)$, i.e., the probability $P\left(\cap_{k=1}^{n}\{N\left(B_{k}\right)=1\cap \left\{N\left(D^{*}\right)=0\right\}\right)/P\left(N(D^{\prime })=n\right)$ has the form

$$\frac{\exp\left(-\int_{D^{\prime }}\lambda \left(x\right)dx\right)\prod_{k=1}^{n}\int_{B_{k}}\lambda \left(x\right)\;dx}{\exp\left(-\left(\int_{D^{\prime }}\lambda \left(x\right)\;dx\right){\left(\int_{D^{\prime }}\lambda \left(x\right)\;dx\right)}^{n}/n!\right.}=\frac{n!\prod_{k=1}^{n}\int_{B_{k}}\lambda \left(x\right)\;dx}{{\left(\int_{D^{\prime }}\lambda \left(x\right)\;dx\right)}^{n}}.$$

Since the *n* Poisson points can be assigned to the subsets $\left\{B_{k}\right\}$ in n! distinct ways (permutations of *n* objects), the above conditional probability has to be scaled by n!, so that it becomes $\prod_{k=1}^{n}\int_{B_{k}}\lambda \left(x\right)\;dx/{\left(\int_{D^{\prime }}\lambda \left(x\right)\;dx\right)}^{n}$. The limit of the latter probability as the subsets $\left\{B_{k}\right\}$ are reduced to points $\left\{\xi_{k}\right\}$ constitutes the density of *n* unordered Poisson points in $D^{\prime }$ given by (13). □

If *N* is a homogeneous Poisson random field with intensity λ(x)=λ, the density in (13) takes the form $f_{\Gamma |(N\left(D^{\prime })=n)\right)}\left(\xi_{1},\ldots ,\xi_{n}\right)=1/{\left(v(D^{\prime })\right)}^{n}$ so that the Poisson points conditional on $N(D^{\prime })=n$ are independent uniformly distributed in $D^{\prime }$.

**Property 5.**  *The mean and correlation functions of Y are*

(14) $$\begin{array}{cc} E[Y(x)] & =E\left[Y_{1}\right]\int_{D^{\prime }}E\left[h\left(R_{1}\left(x-\xi \right)\right)\right]\;\lambda \left(\xi \right)\;d\xi \;\;\mathrm{and}\;\\ \mathrm{Cov}\left[Y\left(x\right)\;Y\left(x^{\prime }\right)\right] & =E\left[Y_{1}^{2}\right]\int_{D^{\prime }}E\left[h\left(R_{1}\;\left(x-\xi \right)\right)\;h\left(R_{1}\;\left(x^{\prime }-\xi \right)\right)\right]\;\lambda \left(\xi \right)\;d\xi . \end{array}$$

**Proof.**  The mean of Y(x) results from the equalities

$$E\left[Y\left(x\right)\right]=E\left\{E\left[\sum_{k=1}^{N\left(D^{\prime }\right)}Y_{k},h\left(R_{k}\left(x-\xi_{k}\right)\right)|N\left(D^{\prime }\right)\right]\right\}$$

and

$$\begin{array}{cc} E\left[\sum_{k=1}^{N\left(D^{\prime }\right)}Y_{k}h\left(R_{k}\left(x-\xi_{k}\right)\right)|N\left(D^{\prime }\right)\right] & =\sum_{k=1}^{N\left(D^{\prime }\right)}E\left[Y_{1}\right]\int_{D^{\prime }}h\left(R_{1}\;\left(x-\xi \right)\;\lambda \left(\xi \right)\;d\xi \right)/\int_{D^{\prime }}\lambda \left(x\right)\;dx\\ \; & =N\left(D^{\prime }\right)\;E\left[Y_{1}\right]\int_{D^{\prime }}h\left(R_{1}\;\left(x-\xi \right)\;\lambda \left(\xi \right)\;d\xi \right)/\int_{D^{\prime }}\lambda \left(x\right)\;dx \end{array}$$

by using the mutual independence of $\left\{Y_{k}\right\}$, $\left\{R_{k}\right\}$ and *N*, the iid property of the random variables $\left\{Y_{k}\right\}$ and of the rotation matrices $\left\{R_{k}\right\}$, and the formula of the density in (13) for n=1. The final expression results from $E\left[N\left(D^{\prime }\right)\right]=\int_{D^{\prime }}\lambda \left(x\right)\;dx$.

The covariance function of *Y* results from the equalities

$$E\left[Y\left(x\right)Y\left(x^{\prime }\right)\right]=E\left\{E\left[\sum_{k,l=1}^{N\left(D^{\prime }\right)}Y_{k}Y_{l}h\left(R_{k}\left(x-\xi_{k}\right)h\left(R_{l}\left(x-\xi_{l}\right)\right)|N\left(D^{\prime }\right)\right]\right\}\right.$$

and

$$\begin{array}{cc} & E\left[\sum_{k,l=1}^{N\left(D^{\prime }\right)}Y_{k}Y_{l}h\left(R_{k}\left(x-\xi_{k}\right)h\left(R_{l}\left(x-\xi_{l}\right)\right)|N\left(D^{\prime }\right)\right]\right.\\ & =\sum_{k=1}^{N\left(D^{\prime }\right)}E\left[Y_{1}^{2}\right]b\left(x,x^{\prime }\right)/E\left[N\left(D^{\prime }\right)\right]+\sum_{k,l=1,k\neq l}^{N\left(D^{\prime }\right)}E{\left[Y_{1}\right]}^{2}a\left(x\right)a\left(x^{\prime }\right)/E{\left[N\left(D^{\prime }\right)\right]}^{2}. \end{array}$$

The above terms are for k=l and k≠l, where $a\left(x\right)=\int_{D^{\prime }}E\left[h\left(R_{1}\left(x-\xi \right)\right)\right]\lambda \left(\xi \right)\;d\xi$ and $b\left(x,x^{\prime }\right)=\int_{D^{\prime }}E\left[h\left(R_{1}\left(x-\xi \right)\right)h\left(R_{1}\left(x^{\prime }-\xi \right)\right)\right]\lambda \left(\xi \right)\;d\xi$. Then, the unconditional expectation of $Y\left(x\right)Y\left(x^{\prime }\right)$ is

$$\begin{array}{cc} E\left[Y\left(x\right)Y\left(x^{\prime }\right)\right] & =E\left[N(D^{\prime })\right]\;E\left[Y_{1}^{2}\right]\;b\left(x,x^{\prime }\right)/E\left[N(D^{\prime })\right]\\ \; & +E\left[N{\left(D^{\prime }\right)}^{2}-N\left(D^{\prime }\right)\right]\;E{\left[Y_{1}\right]}^{2}\;a\left(x\right)\;a\left(x^{\prime }\right)/E{\left[N(D^{\prime })\right]}^{2} \end{array}$$

and since $E\left[N{\left(D^{\prime }\right)}^{2}-N\left(D^{\prime }\right)\right]=E{\left[N(D^{\prime })\right]}^{2}$, we have $E\left[Y\left(x\right)Y\left(x^{\prime }\right)\right]=E\left[Y_{1}^{2}\right]\;b\left(x,x^{\prime }\right)+E{\left[Y_{1}\right]}^{2}\;a\left(x\right)\;a\left(x^{\prime }\right)$ and $\mathrm{Cov}\left[Y\left(x\right)\;Y\left(x^{\prime }\right)\right]=E\left[Y_{1}^{2}\right]\;b\left(x,x^{\prime }\right)$, i.e., the formula in (14). □

The calculations of the first two moments of the random field Y(x) in (14) involve multidimensional integration which, generally, needs to be performed numerically. The expression of these moments simplifies somewhat to

(15) $$\begin{array}{cc} E[Y(x)] & =\lambda \;E\left[Y_{1}\right]\;\int_{D^{\prime }}E\left[h\left(R_{1}\;\left(x-\xi \right)\right)\right]\;d\xi \;\mathrm{and}\\ \mathrm{Cov}\left[Y\left(x\right),Y\left(x^{\prime }\right)\right] & =\lambda \;E\left[Y_{1}^{2}\right]\;\int_{D^{\prime }}E\left[h\left(R_{1}\;\left(x-\xi \right)\right)\;h\left(R_{1}\;(x^{\prime }-\xi \right)\right]\;d\xi \end{array}$$

if *N* is a homogeneous Poisson field with constant intensity λ.

#### 2.2.2. Inclusion Properties

It is common to consider kernels $h:R^{d}\to \left[0,\infty \right)$ which decrease from their largest value h(0)=1 sufficiently fast such that the value of Y(x) in small vicinities $\left\{D\left(\Gamma_{k}\right)\right\}$ of the Poisson points $\left\{\Gamma_{k}\right\}$ is dominated by $\left\{Y_{k}\;h\left(R_{k}\;\left(x-\Gamma_{k}\right)\right)\right\}$. Under these assumptions, $Y\left(x\right)≃Y_{k}\;h\left(R_{k}\;\left(x-\Gamma_{k}\right)\right)$ for $x\in D(\Gamma_{k})$ and the number of inclusions in *D* is in the interval $\left(N\left(D\right),N\left(D^{\prime }\right)\right)$. The latter statement implies that the average number of inclusions in *D* is in the range $\left(E\left[N(D)\right],E\left[N(D^{\prime })\right]\right)$.

These observations imply that the number of inclusions by level-cut filtered Poisson fields can be characterized simply since the probabilities of the expectations of the Poisson variables N(D) and $N(D^{\prime })$ are available analytically. In contrast, even the calculation of the average number of inclusions generated level-cut Gaussian fields is computationally demanding, see (9).

**Property 6.**  *The average of the volume fraction $V_{I}=\int_{D}U\left(x\right)\;dx/v\left(D\right)$ is*

(16) $$E\left[V_{I}\right]=\frac{1}{v(D)}\;E\left[N(D^{\prime })\right]\;E\left[Y_{1}\right]\;\int_{D}E\left[h\left(R_{1}\;\left(x-\Gamma_{1}\right)\right)\right]\;dx.$$

**Proof.**  The characterization of the volume fraction $V_{I}=\int_{D}U\left(x\right)\;dx/v\left(D\right)$ of two-phase material specimens is rather difficult due to the complex dependence of the mean and variance of $V_{I}$ on the mean and correlation functions of *U*. For example, the expectation of the level-cut field *U* is

$$\begin{array}{cc} E[U(x)] & =E\left[\sum_{k=1}^{N(D^{\prime })}Y_{k}\;h\left(R_{k}\;\left(x-\Gamma_{k}\right)\right)\right]=E\left\{E\left[\sum_{k=1}^{N(D^{\prime })}Y_{k}\;h\left(R_{k}\;\left(x-\Gamma_{k}\right)\right)|N\left(D^{\prime }\right)\right]\right\}\\ \; & =E\left\{N\left(D^{\prime }\right)E[Y_{1}\;h\left(R_{1}\;\left(x-\Gamma_{1}\right)\right)\right]\}=E\left[N(D^{\prime })\right]\;E\left[Y_{1}\right]\;E\left[h\left(R_{1}\;\left(x-\Gamma_{1}\right)\right)\right], \end{array}$$

by using properties of the conditional expectation and of the random elements $N(D^{\prime })$, $\left\{R_{k}\right\}$, and $\left\{\Gamma_{k}\right\}$. □

#### 2.2.3. Monte Carlo Algorithm

We have seen that even the expectation of the volume fraction for inclusions generated by level-cut filtered Poisson fields depends on expectation $E\left[h\left(R_{1}\;\left(x-\Gamma_{1}\right)\right)\right]$, which is not available analytically. Numerical methods have to be employed to characterize inclusions produced by these random fields.

A simple Monte Carlo algorithm can be developed for generating synthetic two-phase material specimens in a bounded subset *D* of $R^{d}$ for homogeneous Poisson fields by using the fact that, conditional on their number, the points of these fields are independent and uniformly distributed. If the field is inhomogeneous, the domain *D* can be distorted so that the field becomes homogeneous [9]. The algorithm has the following three steps.

– *Step 1:* Generate independent samples of the Poisson random variable $N(D^{\prime })$, which are integers $\left\{n_{k}\geq 0\right\}$ by employing the methods described in, e.g., [15] (Sect. 4.6).
– *Step 2:* For each sample $n_{k}$ of $N(D^{\prime })$, generate $n_{k}$ independent uniformly distributed sets of points in $D^{\prime }$ and $n_{k}$ independent samples of $\left\{Y_{i}\right\}$ and $\left\{R_{i}\right\}$. Samples of $\left\{Y_{i}\right\}$ and $\left\{R_{i}\right\}$ can be delivered by standard Monte Carlo algorithms. The rejection method can be used to generate $n_{k}$ independent uniformly distributed points in $D^{\prime }$, see [5] (Sect. 2.2).
– *Step 3:* Construct the corresponding realization of Y(x) in (11) and the corresponding level-cut realizations, record cuts above a(x), and estimate statistics of features of the resulting inclusions.

The algorithm can provide complete information on the generated synthetic two-phase materials. The required number of samples of depends on the objective. For example, the estimates of the average number of inclusions require far fewer Gaussian samples than the estimates of, e.g., the geometry of inclusions.

#### 2.2.4. Two-Phase Synthetic Material Specimens

The following illustrations are for d=3 and the kernel $h\left(\xi \right)=\exp\left(-\xi^{\prime }\;\gamma \;\xi \right)$, where γ is a diagonal matrix with non-zero entries $1/\sigma_{r}^{2}>0$, r=1,2,3, and the space-invariant level function a(x)=a. The resulting inclusions are the ellipsoids $\sum_{r=1}^{3}{\left(x-\Gamma_{k}\right)}^{2}/\sigma_{r}^{2}>-\ln\left(a/Y_{k}\right)$ which are generated with probability $P\left(Y_{k}>a\right)$. The following numerical results are for λ(x)=λ=0.1 and $Y_{1}∼10\;\left|N\left(0,1\right)\right|$ is ten times larger than the absolute value of a standard Gaussian variable N(0,1).

**Figure 4:**
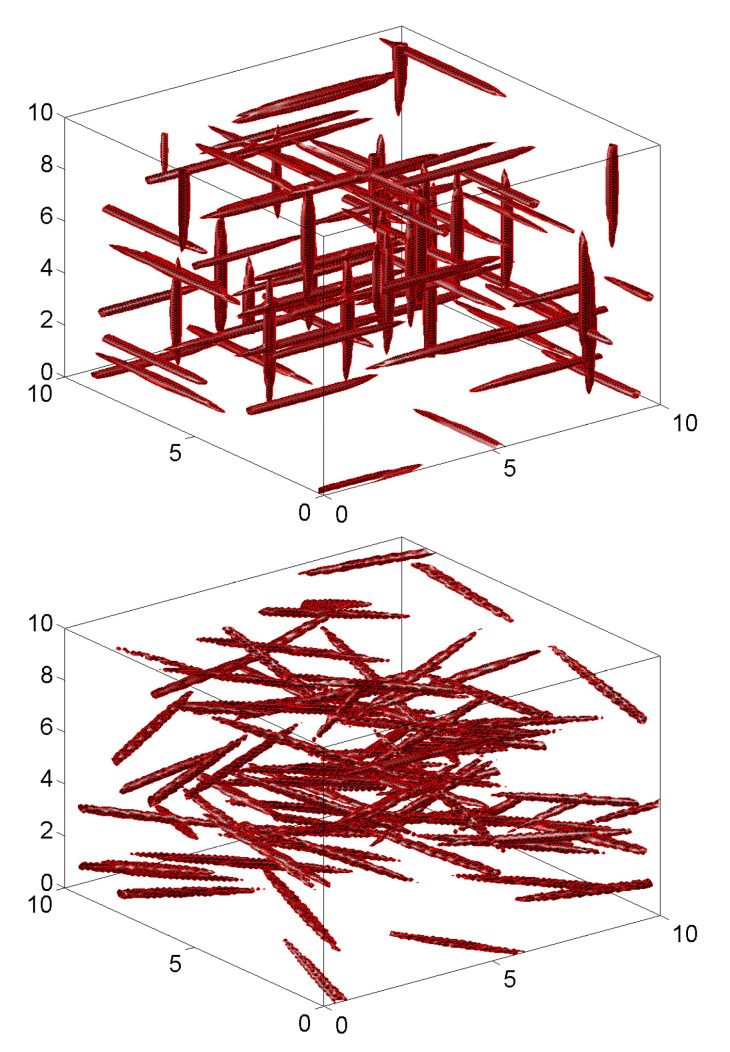
Samples of two-phase microstructures with inclusions of random orientations restricted to the axis of coordinates and random in $R^{3}$ (**top** and **bottom** panels) for $\sigma_{1}=1$, $\sigma_{2}=\sigma_{3}=0.03$, and a=0.01.

The inclusions in Figure 4 are for $\sigma_{1}=1$, $\sigma_{2}=\sigma_{3}=0.03$, and a=0.01 with random orientations along the coordinates of the three-dimensional space. They are elongated since $\sigma_{1}\gg \sigma_{2}=\sigma_{3}$.

**Figure 5:**
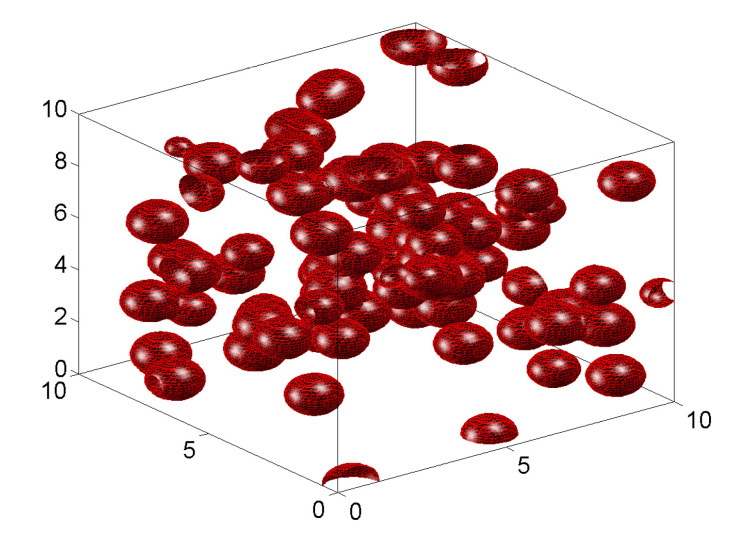

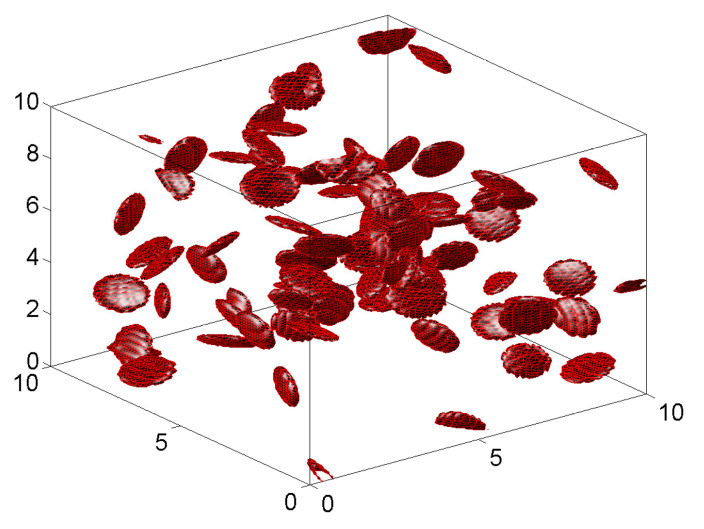
Samples of two-phase microstructures with spherical (**top panel**) and disk (**bottom panel**) inclusions.

The microstructures in the top panels of Figure 5 are for $\sigma_{1}=\sigma_{2}=\sigma_{3}=0.3$. The inclusions are spheres placed at Poisson random points in space. The rotation matrices $\left\{R_{k}\right\}$ are irrelevant in this case. The two-phase specimen in the bottom panel of Figure 5 is for $\sigma_{1}=\sigma_{2}=0.3$ and $\sigma_{3}=0.05$ and random rotations.

These illustrations demonstrate a remarkable versatility of the model. The level-cut filtered Poisson field can generate a broad range of inclusion types by changing scale parameters in the definition of a very simple kernel function.

## 3. Mosaic Fields

Consider as previously a material specimen in a bounded subset *D* of $R^{d}$, d=1,2,3, and a bounded subset $D^{\prime }$ including *D*. The random fields considered here are completely defined by (1) binomial or Poisson point fields and (2) random bounded subsets of $R^{d}$. The binomial and Poisson point fields are unions of deterministic and random numbers of independent uniformly distributed points in $D^{\prime }$.

The following subsections define binomial and Poisson point fields, give essential properties of these fields, present an algorithm for the construction of synthetic two-phase material specimens, and illustrate numerically features of binomial and Poisson-based material specimens.

### 3.1. Binomial Point Fields

Consider *n* independent, uniformly distributed points $\left\{\Gamma_{i}\right\}$, i=1,…,n, in $D^{\prime }\subset R^{d}$. The corresponding binomial point field is the union of these points. Then, the probability that the points of a binomial field belong to a family $\left\{A_{i}\right\}$, i=1,…,n, of disjoint subsets of $D^{\prime }$ is

(17) $$P\left(\cap_{i=1}^{n}\left\{\Gamma_{i}\in A_{i}\right\}\right)=\prod_{i=1}^{n}P\left(\Gamma_{i}\in A_{i}\right)=\prod_{i=1}^{n}\frac{v(A_{i})}{v(D^{\prime })},$$

by the independence of the events $\left\{\Gamma_{i}\in A_{i}\right\}$ and the distribution of the points $\left\{\Gamma_{i}\right\}$, where $v(A_{i})$ and $v(D^{\prime })$ denote the volumes of $A_{i}$ and $D^{\prime }$. Useful information on this process can be found in [5] (Sect. 2.2). Note that the numbers of binomial points in two disjoint subsets *A* and *B* of $D^{\prime }$ are not independent since if, e.g., there are m≤n points in *A*, there can be at most n−m points in *B*.

**Property 7.**  *The average number of binomial points N(A) in a subset A of $D^{\prime }$ is $E\left[N(A)\right]=n\;v\left(A\right)/v\left(D^{\prime }\right)$.*

**Proof.**  *The expectation of the number $N\left(A\right)=\sum_{i=1}^{n}1\left(\Gamma_{i}\in A\right)$ of binomial points in a subset A of $D^{\prime }$ is $E\left[N(A)\right]=\sum_{i=1}^{n}E\left[1\left(\Gamma_{i}\in A\right)\right]=\sum_{i=1}^{n}P\left(\Gamma_{i}\in A\right)=n\;v\left(A\right)/v\left(D^{\prime }\right)$. The second equality holds since $1\left(\Gamma_{i}\in A\right)$ is a random variable taking the values 1 and 0 with probabilities $P\left(\Gamma_{i}\in A\right)$ and $1-P\left(\Gamma_{i}\in A\right)$. The latter equality is valid since the points $\left\{\Gamma_{i}\right\}$ are identically uniformly distributed in $D^{\prime }$. □*

### 3.2. Poisson Point Fields

The Poisson point field is completely defined by the following two properties: (1) the number of points N(A) in an arbitrary bounded subset *A* of $R^{d}$ is a Poisson random variable with intensity $E\left[N(A)\right]$, and (2) the numbers of points $\left\{N\left(A_{i}\right)\right\}$ in disjoint bounded subsets $\left\{A_{i}\right\}$ of $R^{d}$ are independent Poisson variables with intensities $\left\{E\left[N(A_{i})\right]\right\}$. Useful information on this process can be found in [5] (Sects. 4.2 and 4.3).

The defining properties of the Poisson field imply that the probability that there are $\left\{n_{i}\geq 0\right\}$ Poisson points in the disjoint subsets $\left\{A_{i}\right\}$, i=1,…,n, of $D^{\prime }$ has the form

(18) $$P\left(\cap_{i=1}^{n}\left\{N\left(A_{i}\right)=n_{i}\right\}\right)=\prod_{i=1}^{n}P\left(N\left(A_{i}\right)=n_{i}\right)=\exp\left(-\sum_{i=1}^{n}E\left[N(A_{i})\right]\right)\;\prod_{i=1}^{n}\frac{E{\left[N(A_{i})\right]}^{n_{i}}}{n_{i}!}$$

by using the independence of the events $\left\{N\left(A_{i}\right)=n_{i}\right\}$ and the probabilities of the Poisson random variables $\left\{N\left(A_{i}\right)\right\}$.

The probability law of the homogenous Poisson field depends on a single parameter λ>0, which can be interpreted as the average number of points in a subset of $R^{d}$ of unit volume. This parameter becomes a real-valued, non-negative function λ(x), $x\in R^{d}$. Then, λ(x)dx is viewed as the average number of Poisson points in the infinitesimal volume dx and its average number changes with the location of dx.

**Property 8.**  *The average number of Poisson points N(A) in a subset A of $D^{\prime }$ is $E\left[N(A)\right]=\int_{A}\lambda \left(x\right)\;dx$ and $E\left[N(A)\right]=\lambda \;v\left(A\right)$ for inhomogeneous and homogeneous Poisson fields with intensities λ(x) and λ.*

**Proof.**  This follows from the fact that N(A) is a Poisson random variable and the meaning of the Poisson intensity parameters, which give the expectations $E\left[N(A)\right]=\int_{A}\lambda \left(x\right)\;dx$ and $E\left[N(A)\right]=\lambda \;v\left(A\right)$ for inhomogeneous and homogeneous Poisson fields for any subset *A* of $D^{\prime }$, where v(A) denotes the volume of *A*. Properties of a Poisson random variable can be found in [14] (Chap. 4). □

### 3.3. Inclusion Properties

Let $\left(\xi_{1},\ldots ,\xi_{n}\right)$ and $\left(\xi_{1},\ldots ,\xi_{N(D^{\prime })}\right)$ be realizations of the binomial and the Poisson point fields in $D^{\prime }$, i.e., samples of $\left(\Gamma_{1},\ldots ,\Gamma_{n}\right)$ and $\left(\Gamma_{1},\ldots ,\Gamma_{N(D^{\prime })}\right)$. The points $\left(\Gamma_{1},\ldots ,\Gamma_{n}\right)$ are independent and uniformly distributed in $D^{\prime }$ and so are the points $\left(\Gamma_{1},\ldots ,\Gamma_{N(D^{\prime })}\right)$ conditional on $N(D^{\prime })$. Let $\left\{A_{i}\right\}$, i=1,2,…, be independent copies of a set-valued random variable. The mass centers or any other points of the subsets $\left\{A_{i}\right\}$ are selected to coincide with the origin of the system of coordinates. An example is the model in [1], which constructs synthetic concrete aggregates based on a large set of data.

As for the level-cut filtered Poisson fields, we generate binomial/Poisson points in $D^{\prime }⊃D$ rather the specimen domain *D* since subsets $\left\{A_{i}\right\}$ centered outside *D* may intersect *D* such that they would generate inclusions in the synthetic specimen. For example, let $D=\times_{i=1}^{d}\left[a_{i},b_{i}\right]$ be the domain of a rectangular specimen and r>0 be the radius of a disk which is sufficiently large to include the random subsets $\left\{A_{i}\right\}$ with probability one. Then, a subset $A_{i}$ whose mass center is outside $D^{\prime }=\times_{i=1}^{d}\left[a_{i}-r,b_{i}+r\right]$ will not generate an inclusion in *D* since $A_{i}\cap D=\emptyset$, while subsets $A_{i}$ with centers in $D^{\prime }\setminus D$ may generate inclusions in *D*.

We now construct two-phase material models based on the binomial and Poisson point fields. The subsets $\left\{A_{i}\right\}$, which are centered at the origin of the system of coordinates, are translated to the binomial/Poisson points and then rotated by using iid random rotation matrices $\left\{R_{i}\right\}$, i.e., they are mapped to $\left\{{\tilde{A}}_{i}=\Gamma_{i}+R_{i}\left(A_{i}\right)\right\}$. It is assumed that the generated inclusions are unlikely to overlap, i.e., that $P\left({\tilde{A}}_{i}\cap {\tilde{A}}_{j}\right)≃0$, i≠j. The resulting fields, referred to as mosaic or Boolean fields—see [3] (Chap. A) and [5] (Chap. 3), can be defined approximately by

(19) $$A\left(x\right)=\sum_{i=1}^{m}1\left(x\in (\Gamma_{i}+R_{i}\left(A_{i}\right))\right)=\sum_{i=1}^{m}1\left(x\in {\tilde{A}}_{i}\right),\;x\in D,$$

so that A(x)=1 if there is an inclusion at *x* and zero otherwise. The number of terms in the above summation is m=n for the Binomial field and $m=N(D^{\prime })$ for the Poisson field.

**Property 9.**  *The expectations of the (random) volume fraction*

(20) $$V_{I}=\frac{1}{v(D)}\;\int_{D}A\left(x\right)\;dx=\frac{1}{v(D)}\;\sum_{i=1}^{m}\int_{D}1\left(x\in {\tilde{A}}_{i}\right)\;dx=\frac{1}{v(D)}\;\sum_{i=1}^{m}v\left({\tilde{A}}_{i}\cap D\right),$$

*of the binomial and Poisson field synthetic material specimens are*

(21) $$\begin{array}{cc} E[V_{I}] & =E\left[\sum_{i=1}^{n}v\left({\tilde{A}}_{i}\cap D\right)\right]=\sum_{i=1}^{n}E\left[v({\tilde{A}}_{i}\cap D)\right]=n\;E\left[v({\tilde{A}}_{1}\cap D)\right]\;\mathrm{and}\\ E[V_{I}] & =E\left[\sum_{i=1}^{N(D^{\prime })}v\left({\tilde{A}}_{i}\cap D\right)\right]=E\left\{\;E\left[\sum_{i=1}^{N(D^{\prime })}v\left({\tilde{A}}_{i}\cap D\right)|N\left(D^{\prime }\right)\right]\right\}=E\left[N(D^{\prime })\right]\;E\left[v({\tilde{A}}_{1}\cap D)\right], \end{array}$$

*for the binomial and the Poisson fields.*

**Proof.**  The equalities for the binomial field hold by the linearity of the expectation operator and the assumptions that the binomial points $\left\{\Gamma_{i}\right\}$, the rotation matrices $\left\{R_{i}\right\}$, and the subsets $\left\{A_{i}\right\}$ are iid random elements so that $E\left[v({\tilde{A}}_{i}\cap D)\right]=E\left[v({\tilde{A}}_{1}\cap D)\right]$, i≥2.

The expression of $E[V_{I}]$ for the Poisson field holds by the properties of the conditional expectation and the fact that the conditional expectation in the square brackets can be written in the form

$$E\left[\sum_{i=1}^{N(D^{\prime })}v\left({\tilde{A}}_{i}\cap D\right)|N\left(D^{\prime }\right)\right]=\sum_{i=1}^{N(D^{\prime })}\;E\left[v({\tilde{A}}_{i}\cap D)\right]=N\left(D^{\prime }\right)\;E\left[v({\tilde{A}}_{1}\cap D)\right]$$

as shown by arguments used for the binomial field, i.e., the rotation matrices $\left\{R_{i}\right\}$, the subsets $\left\{A_{i}\right\}$, and the Poisson points $\left\{\Gamma_{i}\right\}$ conditional on $N(D^{\prime })$ are iid random elements. The average number of Poisson points in $D^{\prime }$ is $E\left[N\left(D^{\prime }\right)\right]=\int_{D^{\prime }}\lambda \left(x\right)\;dx$ and $E\left[N\left(D^{\prime }\right)\right]=\lambda \left(x\right)\;v\left(D^{\prime }\right)$ for inhomogeneous and homogeneous Poisson fields. □

The expressions of the volume fractions in (21) involve the expectations $\left\{E\left[v({\tilde{A}}_{i}\cap D)\right]\right\}$, which are available analytically only in special cases. These expectations can be estimated from realizations of synthetic specimens produced by Monte Carlo algorithms which generate binomial/Poisson points in $D^{\prime }$, assign subsets $\left\{A_{i}\right\}$ to these points, and rotate them according to the random matrices $\left\{R_{i}\right\}$.

The parameters of the binomial and Poisson fields can be inferred from data on actual specimens. For example, suppose that the inclusion geometry is available so that the expectations $\left\{E\left[v({\tilde{A}}_{1}\cap D)\right]\right\}$ can be calculated. Then, it remains to find the parameter *n* of the binomial field and the parameter λ of the Poisson field assumed to be homogeneous. These parameters can be obtained from the equalities $n=n_{I}$ and $\lambda \;v\left(D^{\prime }\right)=n_{I}$, where $n_{I}$ denotes the average number of inclusions recorded from measurements. If the Poisson field is inhomogeneous, the determination of its intensity function λ(x) requires information beyond the average number of inclusions.

The binomial/Poisson-based fields in this subsection and the level-cut filtered Poisson fields of Section 2.2 are similar in the sense that their inclusions are random sets placed at random locations in $D^{\prime }$. However, the geometry of the inclusions produced by filtered Poisson fields is more restrictive than that of binomial/Poisson fields, which has no constraints.

#### 3.3.1. Monte Carlo Algorithm

We have seen that even the expected volume fraction of synthetic two-phase materials generated by binomial and Poisson fields is difficult to calculate. However, simple Monte Carlo algorithms can be implemented to characterize the inclusions of these materials.

The algorithms are based on (1) the fact that Poisson points, conditional on their number, are independent and uniformly distributed in $D^{\prime }$ and (2) the available methods for generating independent and uniformly distributed in bounded subsets $D^{\prime }$ of arbitrary shapes.

The following three-step Monte Carlo simulation algorithm can be used to construct and characterize synthetic two-phase material specimens in a bounded subset *D* of $R^{d}$.

– *Step 1:* Generate samples $\left\{n_{k}\right\}$ of the Poisson random variable $N(D^{\prime })$ by employing the methods described in, e.g., [15] (Sect. 4.6).
– *Step 2:* For each sample $n_{k}$ of $N(D^{\prime })$, generate sets of $n_{k}$ independent uniformly distributed points in $D^{\prime }$ and $n_{k}$ random sets $\left\{A_{i}\right\}$. There are several methods for generating realizations of binomial points in $D^{\prime }$ [5] (Sect. 2.2). For example, the rejection method generates $n_{R}\geq n_{k}$ independent, uniformly distributed points in a rectangle $R=\times_{i=1}^{d}\left[a_{i},b_{i}\right]$ including the subset $D^{\prime }$ by using, e.g., the MATLAB (R2020) function $a_{i}+\left(b_{i}-a_{i}\right)\;\mathrm{rand}\left(1,n_{R}\right)$, i=1,…,d. Then, it retains from these points those which fall in $D^{\prime }$.
– *Step 3:* Construct the corresponding realization of the mosaic field and estimate statistics of features of the resulting inclusions.

For the binomial fields, the first step is not needed since their number of points *n* is specified so that $n_{k}=n$ in the second step. The algorithm can provide complete information on synthetic two-phase materials but requires different numbers of samples for different objectives. For example, the estimates of the average number of inclusions require far fewer samples than the estimates of, e.g., the inclusion geometries.

#### 3.3.2. Two-Phase Synthetic Material Specimens

The binomial and Poisson fields are applied to generate two-dimensional rectangular two-phase material specimens in D=[0,a]×[0,b], a,b>0. The inclusions are deterministic rectangles with sides $\left(a_{I},b_{I}\right)$ whose bottom left vertices are uniformly distributed in $D^{\prime }$, i.e., they are placed at binomial points. Then, they are rotated counter-clockwise according to the uniform distribution in [0,π/2]. The extended domain is $D^{\prime }=\left[a-r,a+r\right]\times \left[b-r,b+r\right]$, r>0. For the Poisson model, the bottom left vertices of the rectangular inclusions are also uniformly distributed in $D^{\prime }$ conditional on the number $N(D^{\prime })$ of Poisson points in $D^{\prime }$.

**Figure 6:**
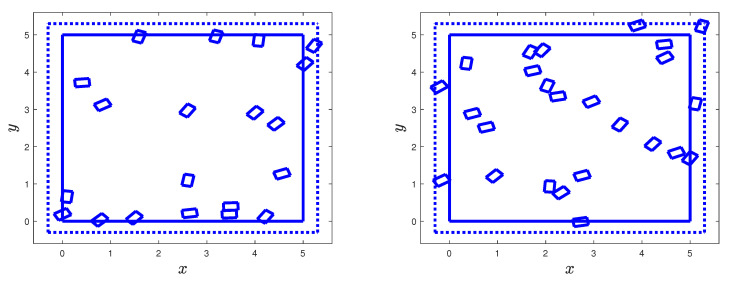
Synthetic two-dimensional, two-phase material specimens with 20 and 25 rectangular inclusions (**left** and **right** panels) of random orientations.

The left and right panels of Figure 6 show samples of the binomial model with n=20 and n=25 inclusions for a=b=5 and r=2. The solid and dotted heavy lines are the boundaries of *D* and $D^{\prime }$. The sides of the rectangular inclusions are $a_{I}=0.3$ and $b_{I}=0.2$.

The left and right panels of Figure 6 can also be viewed as synthetic material specimens generated by the Poisson model under the conditions $N(D^{\prime })=20$ and $N(D^{\prime })=25$. Note also that some of the generated inclusions are partially in *D* and some overlap, a physically impossible event. These observations illustrate difficulties related to the exact determination of the volume fraction for synthetic specimens.

To address the above-mentioned physical inconsistency, the binomial and Poisson models can be modified according to the so-called Mattérn hard-core point process; see [5,7] (Sect. 5.4). The simplest version of this process retains one of the set of binomial/Poisson points closer than a set distance, which is such that the inclusions of the modified point processes do not overlap. For the algorithm used to generate the inclusions in Figure 6, binomial/Poisson points closer than the diagonal of the rectangular inclusions may overlap. It seems that there is an overlap in each synthetic specimens in this figure. The Mattérn hard-core point process will remove one of the overlapping inclusions.

## 4. Tessellation Random Fields

Consider as previously a material specimen occupying a bounded subset *D* of $R^{d}$ and let $\left\{B_{i}\right\}$ be a partition of *D*, i.e., $D=\cup_{i=1}^{m}B_{i}$ where $B_{i}\cap B_{j}=\emptyset$, i≠j, is a union of disjoint subsets. The number *m* of sets $\left\{B_{i}\right\}$ partitioning *D* can be a specified (deterministic) number *n* or an integer-valued, non-negative random variable N(D). The partition of *D* is referred to as tessellation and its members $\left\{B_{i}\right\}$ are called cells.

### 4.1. Voronoi Tessellation

Tessellations have been used to describe microstructures resulting from growth processes occurring during crystallization; see [5] (Sect. 10.2) and [4] (Chap. 8). Aluminum grains, which are regions of aluminum specimens with nearly constant atomic lattice orientations, can be viewed as cells of tessellations [16].

Consider a finite set of points $\left\{x_{i}\in D\right\}$, referred to as centers. The Voronoi tessellation with centers $\left\{x_{i}\right\}$ consists of the disjoint subsets $\left\{B_{i}\right\}$ which include the points of *D* closest to the centers $\left\{x_{i}\right\}$, i.e., $B_{i}=\{x\in D:∥x-x_{i}∥<∥x-x_{j}∥,\;j\neq i\}$. There are various extensions of the Voronoi tessellation, e.g., Delaunay, Johnson–Mehl, and other tessellations, see [5] (Sect. 10.2). As for the previously discussed binomial/Poisson fields, the centers of the Voronoi cells can be placed in a bounded subset $D^{\prime }⊃D$ including the specimen rather than the specimen domain *D*. For simplicity, we generate the centers $\left\{x_{i}\right\}$ in the specimen domain *D*.

The number and the spatial location of the centers $\left\{x_{i}\right\}$ define completely the Voronoi tessellation. For examples, if the components of the centers $\left\{x_{i}\right\}$ are equally spaced along the system coordinates of a rectangular specimen, then the cells $\left\{B_{i}\right\}$ are rectangular. Otherwise, they may have complex geometries. Generally, the number *m* of centers in *D* is a Poisson random variable N(D) with intensity $E\left[N\left(D\right)\right]=\int_{D}\lambda \left(x\right)\;dx$. Then, the tessellation has *n* centers and cells with probability $P\left(N(D)=n\right)=\exp\left(-E\left[N\left(D\right)\right]\right)\;{\left(E[N(D)\right)}^{n}/n!]$, n≥0.

### 4.2. Inclusion Properties

The Voronoi tessellation defines the geometry of a material microstructures. It does not provide any information on the physical/mechanical properties of its cells. It is common to assign attributes to the individual cells independently of the other cells; e.g., a cell $B_{i}$ may be selected to have the properties of phase 1 and 2 with probabilities $p_{1}\in \left[0,1\right]$ and $1-p_{1}$ irrespective of the properties of the cells $\left\{B_{j}\right\}$, j≠i. The resulting two-phase material model is referred to as random checkerboard; see [4] (Sect. 7.2.3) and [6] (Sect. 8.1.3).

**Property 10.**  *The average of the number $N_{1}$ of phase 1 Voronoi cells in D for the binomial and the Poisson Voronoi centers is*

(22) $$\begin{array}{cc} E[N_{1}] & =p_{1}\;n\;\mathrm{and}\\ E[N_{1}] & =p_{1}\;E\left[N(D)\right]. \end{array}$$

**Proof.**  For the binomial model, the number $N_{1}$ of phase 1 Voronoi cells in *D* is the output of a binomial random variable corresponding to *n* independent trials with probability of success $p_{1}$. Then, the average number of phase 1 cells is $E\left[N_{1}\right]=p_{1}\;n$ [14] (Chap. 3).

For the Poisson model, the number of phase 1 Voronoi cells in *D* conditional on N(D) is $E\left[N_{1}|N\left(D\right)=n\right]=p_{1}\;n$. The unconditional expectation of $N_{1}$ has the form

$$\begin{array}{cc} E[N_{1}] & =\sum_{n=0}^{\infty }p_{1}\;n\;P\left(N(D)=n\right)=p_{1}\;\exp\left(-E[N(D)]\right)\;\sum_{n=0}^{\infty }n\;\frac{E{\left[N\left(D\right)\right]}^{n}}{n!}\\ \; & =p_{1}\;E\left[N(D)\right]\;\exp\left(-E[N(D)]\right)\;\sum_{n=1}^{\infty }\frac{E{\left[N\left(D\right)\right]}^{n-1}}{(n-1)!}=p_{1}\;E\left[N(D)\right] \end{array}$$

by noting that $\sum_{n=1}^{\infty }E{\left[N\left(D\right)\right]}^{n-1}/\left(n-1\right)!=\exp\left(E\left[N(D)\right]\right)$. Similar arguments can be used to find higher-order moments of $N_{1}$ for the binomial and the Poisson models. □

We conclude with the observation that the Voronoi checkerboard model is rather restrictive since (1) the cells are polyhedrons whose features are complex functions of the tessellation centers $\left\{\Gamma_{i}\right\}$ so that they can describe inclusions of simple geometries and (2) the assumption that adjacent cells have independent properties is questionable for most materials. A Markov random field has been developed in [17] which relaxes this assumption. The resulting models are fitted to measurements of the crystallographic texture of AA7075 aluminum plates and subsequently used to generate synthetic aluminum specimens.

#### 4.2.1. Monte Carlo Algorithm

As noted in the previous subsection, the characterization of the geometry of the Voronoi cells poses notable difficulties. Numerical methods need to be employed to characterize the inclusions beyond volume fraction. The Monte Carlo algorithms of the previous sections and existing MATLAB functions can be adapted to generate two-phase material specimens.

The following three-step Monte Carlo simulation algorithm can be used to construct and characterize synthetic two-phase material specimens in bounded subsets *D* of $R^{d}$.

– *Step 1:* Generate samples $\left\{n_{k}\right\}$ of the Poisson random variable N(D) by employing the methods described in, e.g., [15] (Sect. 4.6).
– *Step 2:* For each sample $n_{k}$ of N(D), generate sets of $n_{k}$ independent uniformly distributed points in *D* and use the **voronoi** MATLAB function to partition the specimen domain in Voronoi cells centered at the generated Poisson points.
– *Step 3:* Toss a coin for each Voronoi cell and call it phase 1 (inclusion) with probability $p_{1}\in \left[0,1\right]$ and estimate the statistics of features of the resulting inclusions.

The algorithm can provide complete information on the generated synthetic two-phase materials. The required number of samples depends on the objective. For example, the estimates of the average number of inclusions require far fewer samples than the estimates of, e.g., the geometry of inclusions.

#### 4.2.2. Two-Phase Synthetic Material Specimens

**Figure 7:**
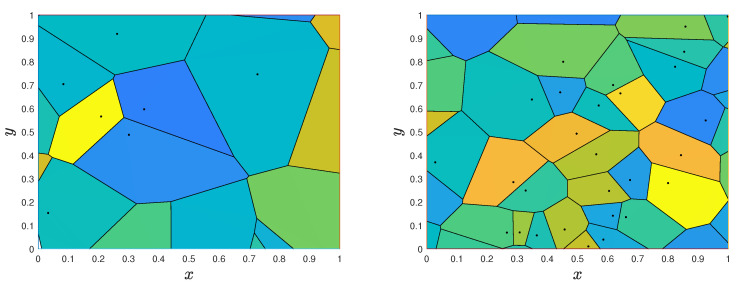
Voronoi tessellation with n=30 and n=100 cells (**left** and **right** panels).

Two realizations of Voronoi tessellations for a two-dimensional specimen $D={\left[0,1\right]}^{2}$ with N(D)=30 and N(D)=100 are shown in the left and right panels of Figure 7. The Voronoi centers are in both figures independent and uniformly distributed in *D*. Different colors are used in different cells to suggest that the Voronoi-based microstructures can be used to represent multi-phase materials.

A notable feature of the Voronoi tessellations is that the phases of the resulting synthetic microstructures do not overlap as, e.g., the inclusions of two-phase materials produced by binomial/Poisson point fields. As noted, a limitation of the model is that the geometry of the generated phases is rather restrictive.

## 5. Conclusions

Level-cut Gaussian/filtered Poisson, binomial/Poisson mosaic, and Voronoi tessellation random fields have been constructed to represent and characterize two-phase random materials. The samples of these fields can be viewed as synthetic two-phase material specimens whose inclusions have random geometries and locations. The manner in which these random fields construct inclusions differs. For example, the inclusions of the level-cut models are defined by values of Gaussian/filtered Poisson fields above specified levels. Mosaic fields place random subsets (inclusions) at binomial/Poisson points. The inclusions of tessellation fields are Voronoi cells selected with some probabilities.

Volume fraction and other properties of the three probabilistic models for two-phase material models have been examined. Monte Carlo algorithms have been presented for generation synthetic material specimens. The presentation includes numerical examples which illustrate the implementation of the material models, clarify some of the theoretical arguments, and illustrate features of these models.
